# Technical Aspects of Patch Reconstruction during Open and Robotic Pancreatoduodenectomy with Venous Resection: Preserving Venous Axis and Collaterals without Sacrificing Radicality

**DOI:** 10.1245/s10434-025-18148-1

**Published:** 2025-09-04

**Authors:** Umberto Cillo, Giampaolo Perri, Enrico Gringeri, Domenico Bassi, Giovanni Marchegiani

**Affiliations:** Hepato-Pancreato-Biliary and Liver Transplant Surgery Unit, Department of Surgical, Oncological and Gastroenterological Sciences (DiSCOG), University of Padua, Padua, Italy

**Keywords:** Pancreatectomy, Vein, Resection, Peritoneal, Falciform, Patch

## Abstract

**Background:**

Different techniques for venous resection and reconstruction during pancreatoduodenectomy are available, each with different advantages and drawbacks.

**Patients and Methods:**

In this multimedia article, a detailed description of the surgical technique of venous resection with peritoneal (falciform) patch reconstruction is provided, including examples of extended (> 5 cm) or low (jejunal veins confluence) venous infiltrations, during both open and robotic pancreatoduodenectomy.

**Results:**

Reconstruction with patch is a versatile technique, typically fit for lateral but cranio-caudally prolonged tumor involvements, which unlike segmental resection allows preservation of venous collaterals, where a simple tangential resection would jeopardize oncologic radicality or increase the risk of stenosis. Moreover, maintaining the original venous axis and direction, it avoids the risk of torsion or kinking potentially associated with segmental resection with or without interposition graft.

**Conclusions:**

The patch reconstruction combines the advantages of both tangential and segmental resection. It can be used for low or extended infiltrations, during open or robotic surgery, preserving venous collaterals without sacrificing radicality, and using prophylactic anticoagulation only.

**Supplementary Information:**

The online version contains supplementary material available at 10.1245/s10434-025-18148-1.

Resection and reconstruction of the portal vein (PV) and/or superior mesenteric vein (SMV) is the standard surgical approach for pancreatic cancer with venous involvement during pancreatoduodenectomy (PD). The International Study Group of Pancreatic Surgery (ISGPS) classifies venous resections into four types, from simple tangential resection with primary closure to complex segmental resection with interposition grafts.^1^ The choice of technique depends on tumor location, extent of vascular involvement, and surgical expertise. While extended resections may improve oncologic outcomes, they carry higher risks, including thrombosis and bleeding. Segmental resections with interposition grafts (type 4), especially allogenic, are linked to increased morbidity and thrombosis, though should be reserved only for extensive, circumferential PV–SMV involvement, where direct anastomosis is not possible.^2–4^ Achieving tension-free end-to-end anastomosis (type 3) often requires significant mobilization and sacrifice of venous collaterals, potentially necessitating total pancreatectomy. However, tangential resections with primary closure (type 1) may compromise margin clearance. A practical alternative is the tangential PV–SMV excision with patch reconstruction (type 2) using autologous tissue, usually from the falciform ligament (as it is covered by peritoneum on both sides).^5, 6^ This approach offers several advantages:

- Immediate graft availability
- Reduced risk of stenosis and better margin clearance compared with primary closure
- Preservation of venous collaterals (including splenic vein with remnant pancreas) compared with segmental resections
- Preservation of venous axis and direction, avoiding the risk of torsion/kinking compared with segmental resections
- No prolonged operative time
- No additional costs
- No need for therapeutic dose anticoagulation
- During robotic resections, it avoids the need of extensive Cattell–Braasch mobilization and to anastomose also the splenic vein^7^

We published a recent series comparing peritoneal patch (*N* = 30) with other types of venous resection for pancreatic cancer, showing how this technique combines the advantages of both tangential and segmental resections—achieving oncologic radicality while preserving venous collaterals—with low morbidity, mortality, and thrombosis rates. The peritoneal patch proved especially useful for low or extended vein involvement and showed outcomes comparable or superior to more complex reconstructions.^8^ At our institution, this method has gained favor also in cases of low (jejunal confluence) or extended (≥ 5 cm) PV–SMV reconstructions and is preferred over segmental resection when feasible, as well as during robotic surgery. This multimedia article provides a comprehensive step-by-step technical description of peritoneal patch reconstruction after PD with PV–SMV resection, including three different case scenarios with video presentations.

## Surgical Techniques

### Selecting the Venous Resection Type

**Fig. 1:**
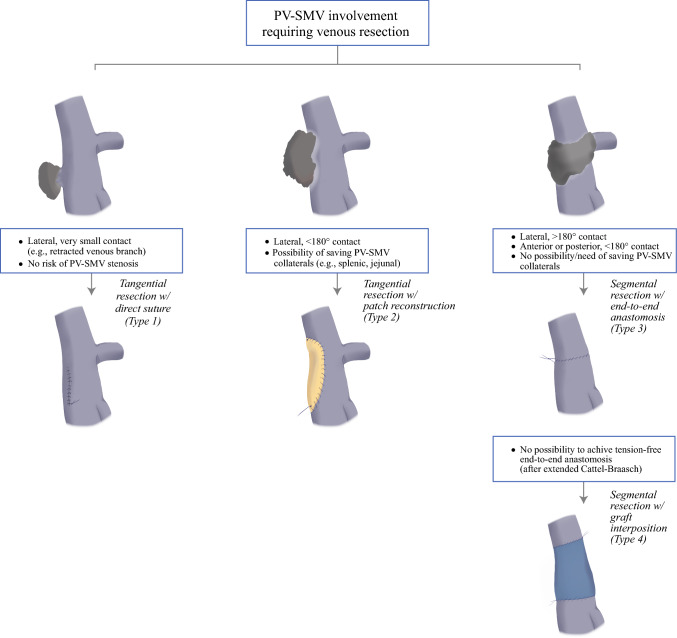
Decisional flowchart for PV–SMV reconstruction techniques

Whenever vascular involvement is preoperatively suspected, an artery-first approach is considered standard for early assessment of possible superior mesenteric artery involvement.^9^ After intraoperative confirmation of PV–SMV involvement, technical feasibility of different resection–reconstruction techniques is evaluated (Fig. 1). In general, tangential resection with primary closure is reserved to very small tumor contact (e.g., ideally ≤ 5 mm, usually requiring incomplete/tangential PV–SMV clamping) with no expected PV–SMV narrowing after repair with direct suture. Segmental resection is used in case of circumferential or antero-posterior (≥ 180°) invasion of the PV–SMV axis. Interposition grafts are reserved to the few cases where tension-free, end-to-end anastomosis is deemed not technically feasible even after a complete Cattell–Braasch maneuver. In all other cases, including low (jejunal veins confluence) or lateral PV–SMV infiltrations extended cranio-caudally (≥ 2 cm), tangential resection with peritoneal patch is preferred to avoid sacrifice of collateral branches of PV–SMV axis.

### Patch Harvesting

The patch usually consists of the autologous falciform ligament, harvested and shaped moments before the venous resection. Falciform ligament has the advantage of being “double-faced” as it is covered by peritoneum on both sides. Alternatively, the patch may be harvested from the peritoneum of the diaphragm or right/left hypochondrium, suturing the peritoneal side inward.

### Venous Resection

The specimen is mobilized from all retroperitoneal attachments except the area of suspected vein involvement. Before resection, the PV–SMV axis is usually marked longitudinally with a surgical skin marker to avoid twisting during reconstruction. The venous resection is performed after complete clamping of PV–SMV axis, including selective clamping of smaller branches to be preserved, and en bloc with the surgical specimen whenever possible. Total clamping time is registered on a stopwatch.

### Patch Reconstruction

**Fig. 2:**
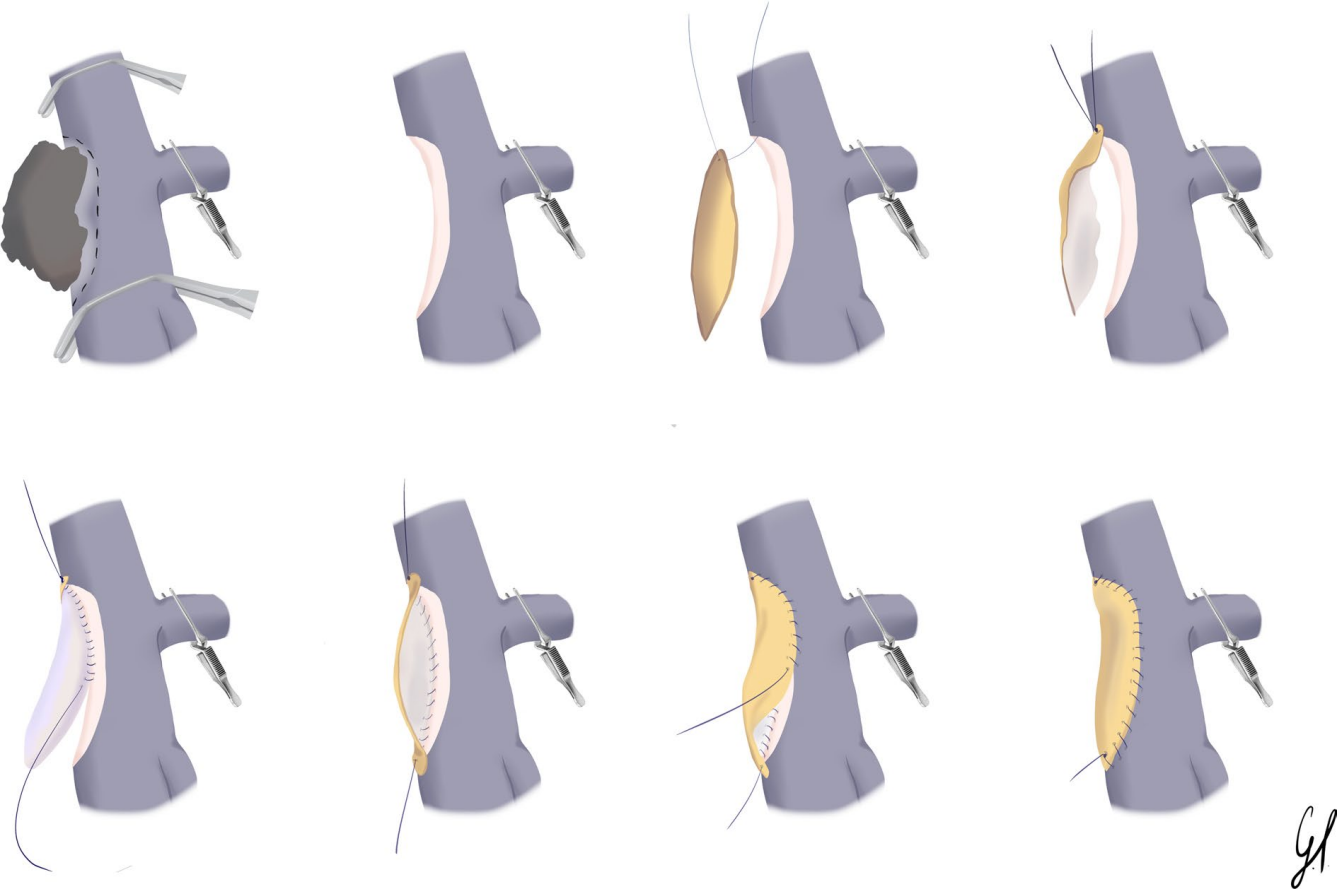
Illustrated surgical technique of peritoneal patch reconstruction

The technique of patch reconstruction is illustrated in Fig. 2. The final venous wall defect is measured with a ruler, and the peritoneal patch is shaped accordingly. A 6-0 double needle polypropylene (PROLENE^™^) suture is placed through the cranial corner of the vein defect (first needle) and through the patch corner (second needle). The patch is then brought into the operative field and approximated to the wall defect, and the suture is tied on the patch side. The first running suture is then performed cranio-caudally, between the right side of the vein and the homolateral patch side. Whenever possible, the suture is passed on the intimal side of the vein only, everting the vein wall to avoid the inclusion of the adventitial connective layer inside the anastomosis. As the caudal corner is reached, the suture is left untied on the patch side, held under tension by a baby clamp. The second running suture is then performed between the left side of the vein and the other patch side. Right before completing the suture, heparinized saline solution is flushed inside the vein. Any eventual surplus of the patch can also be corrected during this phase. The proximal (PV) clamp is then open to distend the patch, and possible bleeding sites are sutured at low pressure. After distal (SMV and collaterals) clamps are removed, and the vein is completely distended, the two ends of the running sutures are then tied together on the caudal corner of the patch side.

## Robotic Technique

The robotic procedure follows the same sequence and steps as the open procedure. In this case, however, the suture consists of two 6-0 single needle expanded polytetrafluoroethylene (ePTFE) (GORE-TEX^®^) sutures, each approximately 15 cm in length, tied together before entering the surgical field (as previously described by other authors^7^). Each needle is passed through the patch first and then through the vein wall. Hence, the running sutures are ready to start. Before portal clamping, however, owing to the slower suture progression compared with the open setting, systemic heparinization may be advisable. Before tying the sutures, the vein is flushed with heparinized saline through an 8 French catheter handled by the table-side assistant. Interestingly, some authors prefer bovine pericardium, which is also readily available in most hospitals worldwide, for patch reconstruction during robotic surgery, as they find it easier to handle robotically—being firmer than the falciform ligament.^10^

### Perioperative Management and Anticoagulation

Low-molecular-weight heparin with prophylactic dose (calculated on the basis of body weight and renal function) is routinely used the day before surgery and postoperatively (for at least 4 weeks) in all patients after peritoneal patch resection. Any variation in the anticoagulation management (from prophylactic to therapeutic) is tailored according to each patient’s possible need for therapeutic anticoagulation, or in case of evidence of postoperative PV–SMV thrombosis. Doppler ultrasound of the liver is routinely performed during the first 48 h after surgery. Contrast-enhanced computed tomography (CT) scan is performed in all patients 30 days after surgery to evaluate PV–SMV patency.

#### Clinical Scenario 1—Extended (> 5 cm) Resection

The first clinical scenario (Supplementary File 1) illustrates PD with extended (> 5 cm) resection of the right lateral aspect of the PV–SMV axis. In this case, tangential resection with primary suture would have been impossible owing to the extension of the infiltration. However, a segmental resection would have required an interposition graft, with the associated risk of thrombosis. The use of falciform patch reconstruction allowed preservation of the splenic vein and the remnant pancreas, the middle colic vein, and the inferior mesenteric vein.

#### Clinical Scenario 2—Low (Jejunal Veins Confluence) Resection

The second clinical scenario (Supplementary File 2) illustrates PD with low resection of the right lateral aspect of the SMV, at the level of the confluence between the 1st and the 2nd jejunal veins. Tangential resection with primary suture would have probably caused stenosis, while end-to-end segmental resection would have been possible only after sacrifice of the first jejunal branch.

#### Clinical Scenario 3—Robotic Resection

The third clinical scenario (Supplementary File 3) illustrates a robotic PD with SMV resection and falciform patch reconstruction. In this particular case, the use of the patch instead of end-to-end reconstruction allowed the avoidance of a complete Cattell–Braasch maneuver, which might be technically complex during robotic PD, and would probably also have required total pancreatectomy.

## Conclusions

The unique advantages of both tangential and segmental venous resections during pancreatectomy can be combined by reconstruction with peritoneal patch. The patch can also be used during robotic surgery, warranting oncologic radicality. Its presence in the reconstruction techniques toolbox ensures the possibility of tailoring the resection according to the level of tumor infiltration, including low or extended lateral infiltrations, without sacrificing the local venous collaterals or the remnant pancreas and maintaining the original axis of the superior mesenteric/portal vein.

## Supplementary Information

Below is the link to the electronic supplementary material.

Supplementary file1 (MP4 159378 kb)

Supplementary file2 (MP4 155782 kb)

Supplementary file3 (MP4 175620 kb)
